# Hidden risks of first-line regimen switching in antiretroviral therapy-treated patients

**DOI:** 10.4102/safp.v67i1.6171

**Published:** 2025-11-14

**Authors:** Shira Kimberley, Mergan Naidoo

**Affiliations:** 1 Department of Haematology, Faculty of Health, Victoria Mxenge Hospital, Durban, South Africa; 2 Department of Family Medicine, Faculty of Public Health, University of KwaZulu-Natal, Durban, South Africa

**Keywords:** people living with HIV, comorbidities, chronic kidney disease, hypertension, antiretroviral therapy

## Abstract

**Background:**

Dolutegravir (DTG) became central to South Africa’s (SA’s) first-line human immunodeficiency virus (HIV) regimen since 2019, offering improved tolerability, fewer drug–drug interactions, and a higher resistance barrier compared to efavirenz (EFV). Concerns remain about the long-term cardiovascular, metabolic, and renal consequences of DTG-based therapy in people living with HIV (PLHIV). This study compared demographic and clinical outcomes across three groups: (1) those on tenofovir, emtricitabine and efavirenz (TEE); (2) those on tenofovir, lamivudine and dolutegravir (TLD); and (3) those switched from TEE to TLD.

**Methods:**

This retrospective, analytical study was conducted at an urban district hospital in KwaZulu-Natal. A sample of 212 patients was calculated using a chi-squared test for multiple proportions (80% power, 95% confidence interval). Data extracted at baseline, 6 and 12 months, were analysed with SPSS version 28.0.

**Results:**

There were statistically significant differences between the three groups. The Switch group showed a higher incidence of new hypertension (HT) (9.9%, *p* = 0.03) and chronic kidney disease (CKD) (12.1%, *p* = 0.01). TLD group maintained stable outcomes. TEE group had the highest incidence of newly diagnosed dyslipidaemia.

**Conclusion:**

DTG-based therapy remains the preferred regimen in SA, its long-term metabolic and renal impact in patients switching from EFV warrants careful surveillance. The increased rates of HT and CKD in the Switch Group emphasises the need for closer monitoring to mitigate against complications.

**Contribution:**

This research contributes to the body of evidence on DTG, highlighting its benefits and the clinical challenges of managing ageing PLHIV with multimorbidity.

## Introduction

Dolutegravir (DTG) stands as a pivotal and highly anticipated breakthrough in the ongoing battle against human immunodeficiency virus (HIV). As the forefront choice for treating HIV infection in the South African National guidelines since 2019,^1^ DTG is set to impact a significant majority of HIV-positive patients, particularly those transitioning from efavirenz (EFV)-based regimens as per the latest 2023 clinical recommendations.^1^ Because DTG is a relatively new drug and is proposed for the use of first-, second- and third-line antiretroviral therapy (ART) regimens, essentially the majority of the South Africa (SA) HIV population on ART will be exposed to this drug at some point in their health-seeking years.^1^

Despite the manifold advantages offered by this innovative drug, there exists a notable gap in our understanding of its long-term clinical implications, particularly regarding potential adverse effects on cardiovascular, metabolic and renal functions in persons living with HIV (PLHIV).^2,3,4,5,6,7,8^ The latest 2023 national guidelines underscore the absence of a direct causal link between DTG and weight gain, attributing this phenomenon to the lower metabolic toxicity of DTG compared to prior ART regimens. While weight gain is acknowledged, it is postulated to stem from the discontinuation of higher toxicity drugs, the ‘return-to-health’ phenomenon and lifestyle influences in an obesogenic environment, with a possible risk factor for weight gain may not be the initiation of DTG itself, but rather due to the switching of drugs from efavirenz to dolutegravir.^1,9,10^

Another documented effect of DTG is its impact on the tubular secretion of creatinine, leading to elevated creatinine without affecting glomerular filtration. The observed rise in serum creatinine concentrations, ‘less than 15%’ in the early stages of treatment, stabilises throughout therapy and is not indicative of discontinuing DTG.^1,11,12,13^ However, the question pertains to the potential contribution of this so-called ‘DTG-effect’ on creatinine levels to the development of chronic kidney disease (CKD) during long-term use or its role as a predictor of future renal complications.^14^

In the pursuit of a comprehensive understanding of DTG’s use in clinical practice, this article, while not discrediting the significant benefits of DTG in terms of rapid virological suppression and low side-effect profile,^1,15,16,17^ explores the gaps in knowledge surrounding its long-term clinical outcomes, shedding light on potential adverse effects on cardiovascular, metabolic and renal functions in PLHIV. Additionally, we delve into the nuanced considerations of weight gain, creatinine dynamics and the broader implications for both treatment-naïve and experienced participant populations, aiming to provide valuable insights for the ongoing refinement of HIV treatment strategies. This study aimed to compare the clinical outcomes of PLHIV with those of different first-line ART regimens at a district hospital in eThekwini, KwaZulu-Natal, with the specific objectives of comparing the demographic and clinical profile of PLHIV on an EFV versus a DTG-based first-line ART regimen and additionally to make recommendations on how the clinical practice at the district hospital may be improved.

## Research methods and design

### Study design and setting

This study employed a retrospective observational analytical cross-sectional design. The study was conducted at the Masibambisane HIV outpatient clinic in Wentworth Hospital, situated in the urban district of the eThekwini health district in KwaZulu-Natal, SA. The clinic provides services to over 1000 PLHIV monthly.

### Study population and sampling strategy

A biostatistician determined a sample size of 212 participants, calculated using a chi-squared test for multiple proportions (80% power, 95% confidence interval). This was assigned proportionately to each group as follows:

Three groups of participant populations were studied: Group 1 included participants initiated on tenofovir, emtricitabine, and efavirenz (TEE Group) and who remained on TEE; Group 2 comprised participants initiated on tenofovir, lamivudine, and dolutegravir (TLD Group) and who stayed on TLD and Group 3 involved participants electively switched from TEE to TLD (Switch group).

The overall number of study participants who met the inclusion criteria was 473, comprising 201 individuals in the TEE group (42.5% of the total 473), 106 in the TLD group (22.4%) and 166 in the Switch group (35.1%). These percentages reflect each group’s representation within the total eligible population. To ensure the final sample of 212 participants accurately represented the population being studied, participants were proportionally allocated according to these group distributions. Accordingly, 90 participants (42.5% of 212) were selected for the TEE group, 48 participants (22.4% of 212) for the TLD group and 74 participants (35.1% of 212) for the Switch group.

**FIGURE 1:**
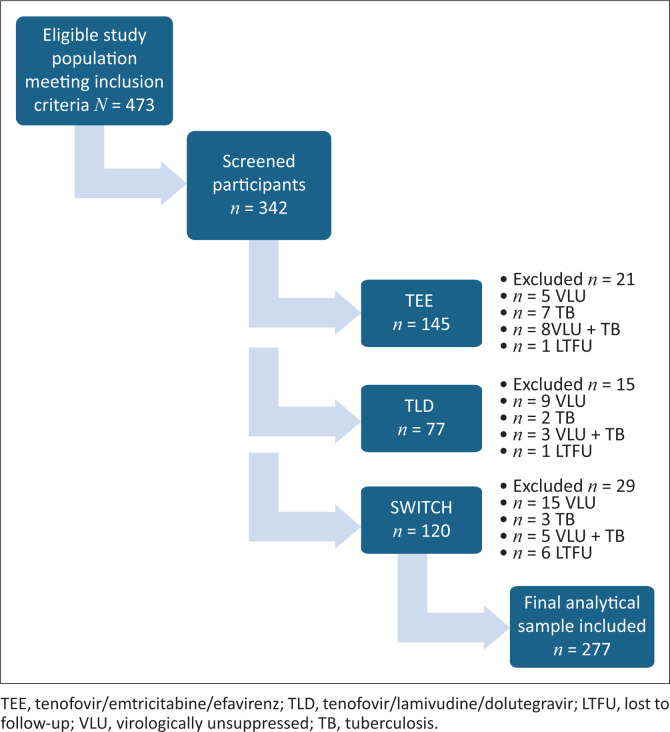
Flow diagram of sample selection.

During manual data collection for the initial sample size of 212 participants, it was identified that some clinical parameters were missing or inadequately documented in a subset of 65 patient records (34 from TEE, 14 from TLD and 17 from the Switch group). To preserve the statistical power and integrity of the analysis, an additional 65 participants were randomly selected using proportional allocation based on the original group distribution. This ensured that each treatment group remained representative of the total population meeting inclusion criteria. By compensating for incomplete data through this method (see Figure 1), the final analysable sample was increased to 277 participants, thereby minimising potential bias and enhancing the reliability of the study findings.

A pilot study involving 10% of the sample size was conducted to assess the feasibility of the data collection tool. Following validation, no adjustments were deemed necessary, as the parameters collected adequately addressed the study’s aim and objectives. The pilot dataset was excluded from the final analysis.

Exclusion criteria, which aimed to eliminate factors that could erroneously alter the desired comparisons, included individuals on first-line ART for less than 12 months, those undergoing treatment for active tuberculosis (TB) infection, pregnant participants, individuals with underlying liver disease and those with documented virological failure (viral load [VL] > 50 at follow-up). Sampling was systematic, employing the K-th sampling method to minimise bias.

### Data collection and analysis

The electronic TIER system was utilised to identify participant files that met inclusion and exclusion criteria. Relevant information was retrospectively collected from participant files and recorded in a data collection tool developed in Microsoft Excel. The tool captured demographic, anthropometric and clinical data from participant’s clinical records, focusing on investigations conducted at baseline visits and subsequent six and 12-month follow-ups.

Definitions for collected data were based on South African national standard treatment guidelines and essential medicines list.^18,19^ In line with Kidney Disease: Improving Global Outcomes (KDIGO), we defined CKD as structural or functional kidney damage persisting for more than 3 months. Parameters included persistent proteinuria (> 2+ dipstick), elevated serum creatinine or estimated glomerular filtration rate (eGFR) < 60 mL/min/1.73 m^2^. The data collected were coded and analysed using SPSS Statistics for Windows, version 28.0 (IBM Corp. 1989, 2021). Categorical data were summarised using percentages and frequencies, while numerical data were presented as mean, median and standard deviation where appropriate.

### Ethical considerations

Ethics approval was obtained via the University of KZN Biomedical Research Ethics Committee (BREC), reference number BREC/00004286/2022. Confidentiality was ensured by encoding all data into numerical values and storing on a password-protected computer.

## Results

In the TEE group, excluded were five with their VL unsuppressed, seven with active TB, eight with unsuppressed VL and TB and one was lost to follow-up (LTFU). The TLD group excluded nine with their VL unsuppressed, two with TB, three with unsuppressed VLs and TB and one LTFU and the Switch group excluded 15 with their VL unsuppressed, three with TB, five with unsuppressed VL and six were LTFU.

The mean age distribution among the three groups was 46, 43 and 48 years, respectively. The racial distribution was predominantly African (91.1%, 91.9%, 90.1%), with the coloured and Indian populations collectively representing less than 10%. The Switch group was noted to be predominantly male (56%), while females predominantly chose to remain on the TEE-based regimen, with a total of 71%, as well as the TLD group showing a female preponderance of 59.7%. The clinical profile among the three groups showed pre-existing comorbidities before ART exposure in the TEE and TLD groups and after exposure to TEE in Switch. Obesity, hypertension (HT) and type 2 diabetes mellitus (T2DM) were noted to be most prevalent in TEE See Table 1, DL and CKD in Switch at baseline (i.e. in those who were TEE exposed) (see Table 1).

**TABLE 1:** Demographics and clinical parameters of the 277 people living with human immunodeficiency virus studied.

Variables	TEE (*N* = 124)		TLD (*N* = 62)		Switch (*N* = 91)		*p*
	*n*	%	*n*	%	*n*	%	
Mean age (years) (s.d.)	46	10.77	43	10.76	48	-	-
**Race**	-	-	-	-	-	-	0.700
African people	114	91.90	57	91.90	82	90.1	-
Indian people	0	0.00	0	0.00	1	1.1	-
Coloured people	10	8.10	5	8.10	8	8.8	-
**Sex**	-	-	-	-	-	-	0.000
Male	36	29.00	25	40.30	51	56.0	-
Female	88	71.00	37	59.70	40	44.0	-
**Pre-existing comorbidities**	(Prior to ART initiation)		(Prior to ART initiation)		(Prior to Switch – i.e. EFV exposed)		-
HT	49	39.50	16	25.80	27	29.7	0.500
T2DM	27	21.80	7	11.30	12	13.2	0.300
DL	13	10.50	2	3.23	19	20.9	0.018
Obesity	49	39.50	13	21.00	25	27.5	0.012
CKD	1	0.80	1	1.60	2	2.2	0.020

Note: The duration on ART (mean in months) for TEE is 69 (standard deviation [s.d.] ± 24); for TLD is 24 (s.d. ± 5) and for Switch is 23 (s.d. ± 7).

HT, hypertension; T2DM, type 2 diabetes mellitus; DL, dyslipidaemia; CKD, chronic kidney disease; TEE, tenofovir/emtricitabine/efavirenz; TLD, tenofovir/lamivudine/dolutegravir; ART, antiretroviral therapy; s.d., standard deviation.

The incidence of newly developed comorbid conditions with statistical significance was observed in the Switch group, with rates of 9.9% (*p* = 0.03) for HT, 20.9% (*p* = 0.001) for obesity and 12.1% (*p* = 0.01) for CKD, respectively. In contrast, the TEE group showed a new onset of dyslipidaemia (DL) at 19.35% (*p* = 0.016). The TLD group remained relatively static.

Eight of the eleven with new onset CKD initially had an early increase in serum creatinine levels after initiating DTG but no return to normal baseline functioning thereafter.

All continuous variables are represented with mean and s.d. Categorical data – used a Pearson’s chi-square analysis (or Fisher’s exact where conditions for chi-square are not met) to determine whether there is a relationship between the worsening variable and group.

### Hypertension

**FIGURE 2:**
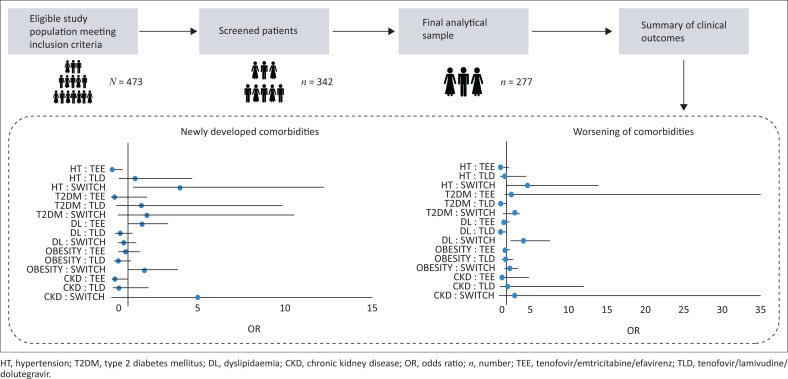
Summary illustration of main findings and forest plot indicating odds ratios for incidence of developing new comorbidities vs the worsening of underlying comorbid control in the three different ART groups.

When assessing all parameters used to determine HT control, compared to the other treatment groups, a significant proportion of those in Switch experienced a statistically significant worsening in HT control – from baseline to 12 months, Fisher’s exact = 11.711, *p* = 0.002 (which is demonstrated predominantly in the development of target organ disease [TOD] upon follow-up). However, looking at the percentage of participants with uncontrolled HT at baseline in comparison to follow-up, the TEE and TLD groups show a marked decrease in uncontrolled blood pressure or improved control (see Figure 2).

### Type 2 diabetes mellitus

There is a significant increase in HbA1c from baseline to 12 months for those in the TEE group (*t* [27] = −3.575, *p* = 0.001) and Switch (*t* [14] = −8.107, *p* < 0.001), with no significant change seen in the TLD group. Interestingly, the TLD group demonstrated improvement in diabetic control upon follow-up visits (see Figure 2).

### Dyslipidaemia

Within all three groups: the results show that there is a significant worsening for all groups, but there is no significant difference in the change between groups in both LDL and triglyceride control.

### Obesity

For all three groups, there is a significant increase in weight from baseline to 12 months; however, this is statistically significant in comparison among the three groups, with TEE showing the most significant weight gain *p* = 0.009, with the largest increase in participants with both grade 2 and 3 obesity. The Switch group showed a large increase in grade 1 obesity and less so in grade 2 and 3 obesity. Tenofovir, lamivudine, and dolutegravir demonstrated the most weight-neutral among all grades of pre-existing obesity.

### Chronic kidney disease

All three groups result in a lower creatinine clearance (CrCl) across time; however, the Switch shows more severe worsening than the other groups, *p* ≤ 0.001. The decrease in CrCl seen in TEE and TLD is still within physiologically accepted normal ranges, but in the Switch group, the mean CrCl drops into the pathological measurement and persists at the 12-month follow-up. Of note, the deterioration in CrCl in Switch is seen among participants newly diagnosed with CKD, as opposed to TEE and TLD in which the CrCl clearance deterioration is also noted among those with pre-existing CKD.

Overall, the TLD group demonstrated a more weight-neutral effect as well as less lability in DM and HT control in participants already known with these diseases.

The incidence of newly developed comorbid conditions with statistical significance was that of HT, obesity and CKD in the Switch group of 9.9% (*p* = 0.03), 20.9% (*p* = 0.001) and 12.1% (*p* = 0.01), respectively, with new onset DL in the TEE group being 19.4% (*p* = 0.016). The TLD group remained relatively static. When looking at control of underlying participant comorbidities (details summarised in Table 2), significant deterioration of pre-existing comorbidities, specifically HT, T2DM and CKD, was noted in the Switch group. Worsening of underlying DL is seen with the greatest OR (see Figure 2) in the Switch group, but this was not of statistical significance in comparison to the TEE or TLD group.

**TABLE 2:** Incidence of newly developed comorbidities across the three groups.

Comorbidities	TEE (*N* = 124)		TLD (*N* = 62)		Switch (*N* = 91)		*p* (Mann–Whitney test)
	*n*	%	*n*	%	*n*	%	
HT	1	0.8	4	6.5	9	9.9	0.030
T2DM	1	0.8	2	3.2	3	3.3	0.610
DL	24	19.4	8	12.9	15	16.5	0.016
Obesity	18	14.5	8	12.9	19	20.9	0.001
CKD	3	2.4	2	3.2	11	12.1	0.010

HT, hypertension; T2DM, type 2 diabetes mellitus; DL, dyslipidaemia; CKD, chronic kidney disease.

Baseline CD4 counts did not vary among the three groups; however, upon follow-up, the DTG-containing groups (Groups 2 and 3) showed a significant increase in CD4 count compared to the TEE group (*p* < 0.001) (see Table 4).

Hospital admissions related to HIV-associated complications were most prevalent in the TEE group (25.5%), 13.3% in the TLD group and 2.7% in the Switch group, while admissions related to comorbid complications were most prevalent in the Switch group (25.4%) compared to the TEE group (6.4%) and the TLD group (17.7%).

Human immunodeficiency virus -associated complications evaluated were cryptococcal meningitis, pneumocystis pneumonia and chronic gastroenteritis/C-difficile. Comorbid associated complications assessed were diabetic ketoacidosis (DKA), cerebrovascular accident (CVA), congestive cardiac failure (CCF), Hypertension (HT) urgency and ischaemic heart disease (IHD).

## Discussion

### Demographics

The representation of the various ethnic groups of participants in each cohort demonstrated no significant statistical difference in keeping with the population being studied. The mean age of participants was also relatively constant among the groups, with most participants in their fourth decade. Although not the objective of this study, this may signify the increasing life expectancy of participants living with HIV and demonstrate the success of South Africa’s ART programme with predominantly older PLHIV.

The sex of participants in the different groups showed a statistically significant difference, with women being more likely to prefer a TEE-based regimen. This may represent a developing stigma being attached to TLD regarding weight gain and perceived teratogenicity already well described in current literature, and thus, further investigation focussing on participant concerns regarding TLD will be beneficial.^20,21^

### Comorbidities

Underlying medical comorbidities prior to ART initiation (most significantly obesity in the TEE group and DL in Switch) were noted and taken into consideration when interpreting the results of the study. The crux of the study focused on analysing the metabolic disease profile of participants in each group over 12 months. In keeping with the investigators’ concerns about the cardio-metabolic effects of DTG,^2,3,4,5,6,7,8,9^ the study noted that there was a statistically significant incidence of newly diagnosed chronic conditions emerging among participants in the Switch group who previously had TEE exposure, being new onset HT and CKD of 9.9% (*p* = 0.03) and 12.1% (*p* = 0.01), respectively.

No statistical difference was noted among the groups with regard to the incidence of newly diagnosed T2DM; however, the Switch group displayed significantly poor control of underlying disease in the rate of increase seen in the HBA1c specifically. This has been previously described in a 2020 study conducted in the United States (US) (see Table 3).^22^ However, the greatest number of participants newly diagnosed with T2DM was seen in the Switch group. Although not statistically significant, a retrospective cohort study in 2020 of 1118 participants did find a 6-9% incidence of moderate hyperglycaemia (7 mmol/L – 13.9 mmol/L) and 1% – 2% incidence of severe hyperglycaemia (> 13.9 mmol/L) after integrase strand transfer inhibitor (INSTI) therapy initiation, with an overall incidence of 5% newly diagnosed T2DM compared to the non-INSTIs incidence of 2%. In the population that transitioned from EFV to an INSTI-based regimen, HBA1c had a statistically significant increase from a mean of 6.4% to 6.9.^22^

**TABLE 3:** Worsening control of pre-existing comorbidities.

Comorbidities	TEE (*N* = 124)				TLD (*N* = 62)				Switch (*N* = 91)				*p*
	At baseline prior to ART initiation		At 12-month follow-up		At baseline prior to ART initiation		At 12-month follow-up		At baseline prior to Switch (EFV exposed)		At 12-month follow-up (post-Switch to DTG)		
	*n*	%	*n*	%	*n*	%	*n*	%	*n*	%	*n*	%
HT	49	-	50	-	16	-	20	-	27	-	36	-	0.002
SBP/DBP>140/90	-	44.9	-	24.1	-	50.0	-	40.0	-	29.6	-	25	-
HT urgency/emergency	-	4.1	-	2.0	0	-	-	5.0	-	3.7	-	13.9	-
Evidence of TOD	-	4.1	10.0	-	-	6.2	-	20.0	-	7.4	-	41.7	-
T2DM (n%)	27	21.8	28	22.6	7	11.3	9	14.5	12	13.2	15	16.5	-
Mean HBA1c g% (s.d.)	6.6	1.3	8.1	1.6	9.6	1.5	8.6	1.4	6.7	0.5	9.7	1.6	0.001
DKA/hyperglycaemic state	1	-	2	-	0	-	1	-	1	-	5	-	1
DL	13	10.5	37	29.8	2	3.2	10	16.1	19	20.9	34	37.4	-
Mean LDL in mmol/L (s.d.)	2.5	0.4	3.2	0.5	2	0.4	2.5	0.3	2.6	0.5	3.4	0.3	0.256
Mean Trigs in mmol/L (s.d.)	1.5	0.4	1.7	0.4	1.3	0.3	1.5	0.2	1.5	0.5	1.8	0.6	0.665
Obesity	49	39.5	67	54.0	13	20.9	21	33.9	25	27.5	44	48.4	-
Grade 1 (BMI 30–34.9)	20	-	30	-	8	-	12	-	13	-	25	-	-
Grade 2 (BMI 35–39.9)	17	-	20	-	3	-	6	-	9	-	12	-	-
Grade 3 (BMI > 40)	12	-	17	-	2	-	3	-	3	-	7	-	-
Mean weight in kg (s.d.)	74.5	18.6	86.1	18.4	72.3	16.3	79.9	17.1	72.3	16.3	79.9	17.1	0.009
CKD	1	0.8	4	3.2	1	1.6	3	4.8	2	2.2	13	14.3	-
Mean CrCl in ml/min (s.d.)	108.8	10.6	100.1	9.6	119	7.1	107.9	8.5	115.6	7.8	95.8	5.6	< 0.001

CKD, chronic kidney disease; BMI, body mass index; T2DM, type 2 diabetes mellitus; HT, hypertension; s.d., standard deviation; HBA1c, heaemoglobin; ART, antiretroviral therapy; EFV, efavirenz; SBP, systolic blood pressure; DBP, diastolic blood pressure; TOD, target organ disease; DKA, diabetic ketoacidosis; LDL, low-density lipoprotein; Trigs, triglycerides; CrCl, creatinine clearance.

The mean increase of HBA1c documented was 3% in the Switch group. Meanwhile, in the TEE group, the mean increase in HBA1c was only 1.5%, showing a relatively neutral effect of EFV on glycaemic control in participants already diagnosed with T2DM. Dolutegravir is known to increase serum metformin concentration, leading to deranged glucose metabolism and necessitating an adjustment in the metformin dose when used concomitantly with DTG.^1^ Further studies are warranted to evaluate this finding. The interesting finding of a mean drop in HBA1C (improved glycaemic control) among the TLD group of participants also requires further evaluation, possibly implying a positive metabolic effect in this group. A study published in the *Southern African Journal of HIV Medicine*, looking at 785 participants between 2011 and 2015 on first- and second-line ART regimens (excluding DTG use), reported that ART was not associated with the occurrence of cardiometabolic disease but rather that BMI was the most significant predictor of the onset of T2DM with an incidence of 5.5%.^2^

The most significant incidence of newly diagnosed DL was in the TEE group. Overall, DL was noted to increase in the EFV-based regimens, in keeping with established literature on lipid elevation associated with EFV.^4,5^ Data summarised from the SPRING-1, SPRING-2, SINGLE and FLAMINGO clinical trials found that DTG had a broadly neutral effect on the overall lipid profile in treatment-naïve participants over 1 year, irrespective of the additional ART drugs used in the treatment regimen.^4^ However, EFV-based ART has been noted to have an association with DL, with a prospective cross-sectional study in 2015 finding a prevalence of metabolic complications, specifically DL, at 47%.^5^ A 2015 study^4^ showed a minor increase in total cholesterol levels with the use of INSTIs (DTG and raltegravir) yet recognised the need for an expanded investigation.

Interestingly, in participants with established underlying comorbidities, TEE group participants demonstrated a statistically significant increase in the severity of obesity. The TEE group showed the most substantial worsening of weight gain, *p* = 0.009, with the most significant increase in participants with both grade 2 and grade 3 obesity. The Switch group showed a significant increase in grade 1 obesity and a lesser increase in grades 2 and 3 obesity. Tenofovir/lamivudine/dolutegravir demonstrated the most weight-neutral among all grades of pre-existing obesity (see Table 3). Further studies to assess EFV and its effect on weight would be beneficial in this regard, as well as subsequent participant education regarding weight gain on different ART regimens, to contribute to the national transition of PLHIV onto DTG-based regimens. It is essential to consider these diseases when evaluating them after ART exposure and the already known genetic suppression EFV has on weight.

In contrast, recent studies have shown that a significantly greater weight gain is observed among the INSTI population compared to EFV, with 31.9% of the study population experiencing a weight gain of more than 5% from baseline, compared to just 20.5% in the non-INSTI group. This was more prominent among females and the black population.^10,22^ A subsequent study in 2017 from Nashville^9^ showed that those participants who switched drug regimens to one containing DTG had the most significant weight gain compared to those remaining on an EFV-based regimen.

New-onset CKD was most significantly seen in the Switch group; additionally, this group showed the most significant deterioration in underlying pre-existing CKD. Furthermore, eight of the eleven with new-onset CKD initially displayed the so-called ‘DTG effect’, characterised by an early but transient increase in serum creatinine levels after initiating DTG, followed by no return to normal baseline functioning thereafter, as expected and described in the current literature.^1,11,12,13^ This may pose a new area of study into whether early yet significant rises in serum creatinine levels when switched from EFV-containing regimens to DTG-based regimens may be a positive predictor of future progression or independently increase the underlying risk of progression to CKD, as this effect was not seen in TLD who were exposed to DTG de novo.

Kidney disease is known to be ubiquitous in the HIV population, whether that be secondary to HIV infection itself, as HIV-associated nephropathy and HIV-associated immune complex kidney disease or as a nephrotoxic side effect brought on by the use of ART. A lack of evidence exists on DTG and its effect on kidney disease (new onset or progression of underlying disease). No significant effect on renal function has been noted in healthy subjects and those with underlying renal impairment.^10^ However, a study out of Portugal in 2021^14^ reported a case of acute interstitial nephritis, later confirmed on a kidney biopsy, with the underlying perpetrator believed to be DTG.

The above data suggest the adverse metabolic effects are predominantly seen when switching participants from a TEE-based regimen to a DTG-based regimen. Evidence of DTG contributing to the progression of underlying pre-existing non-communicable disease (NCD) was seen more so in the Switch group than in the TLD group or the expected natural progression of these diseases in participants exposed to other first-line ART regimens.

### Hospital admissions

**FIGURE 3:**
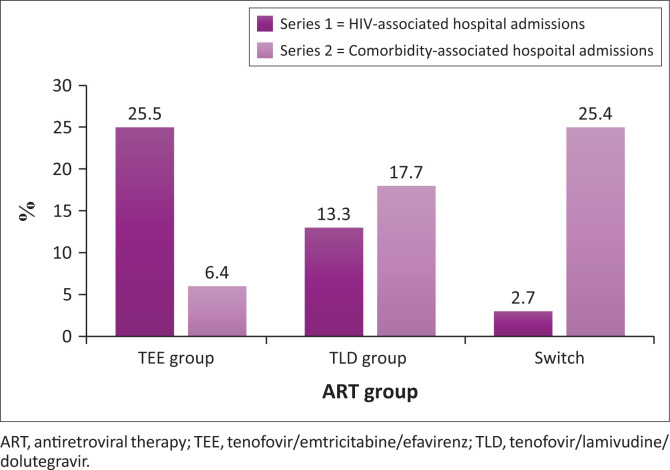
Hospital admissions across the three groups.

Among the different groups, the TEE group demonstrated the highest incidence of admissions related to HIV-associated diseases (opportunistic infections). A total of 30 hospital admissions in TEE, 80% were because of HIV-associated complications (see Figure 3). Groups 2 and 3 participants with DTG exposure also showed a remarkable increase in their CD4 counts, averaging 328 cells/mm^3^, whereas the TEE group had an average increase of only 189 cells/mm^3^ (see Table 4). Postulations for these findings of poor primary HIV control on TEE-based regimens include poor compliance because of poorly tolerated side effects and resistance to EFV among the population being studied. This further reiterates the known need to shift away from EFV-based regimens.^1^

**TABLE 4:** Summary of CD4 count changes at follow-up.

Changes in CD4 count (mean cells/mm^3^)	TEE (*N* = 124)		TLD (*N* = 62)		Switch (*N* = 91)		Kruskal-Wallis
	*n*	%	*n*	%	*n*	%	
Baseline	349	± 85	316	± 55	322	± 86	0.00
12 months	538	± 53	674	± 42	620	± 89	-

Note: The % increase for TEE is 35.1%; for TLD is 53% and for Switch is 48%.

TEE, tenofovir/emtricitabine/efavirenz; TLD, tenofovir/lamivudine/dolutegravir.

This may pose an additional independent risk factor for developing cardiovascular and metabolic complications. A systematic review and meta-analysis of 32 publications between 2000 and 2017 involving 383 471 HIV-positive individuals and more than 798 424 HIV-negative individuals found that the HIV-positive group on ART was more likely to have a myocardial event, with the risk increasing per year of exposure to ART. In contrast, the incidence of a myocardial event was not significantly higher in the HIV-negative group compared to the HIV-positive but ART naïve group.^6^

The Switch group demonstrated a statistically significantly higher number of admissions related to comorbidities such as HT, obesity and CKD (metabolic syndrome) (see Figure 3). These findings, specifically those pertaining to participants with underlying comorbidities who were electively transitioned to a DTG-containing regimen, are worrisome as this may have implications for the long-term metabolic outcome and subsequent additional healthcare burden. This further stresses that the metabolic adverse effects seen post-transition of participants from a TEE to a DTG-based regimen are associated with a statistically significant increase in related hospitalisations. This not only has a public health impact but also a cost impact on the already-strained public healthcare system.

### Strengths and limitations

This study was limited to the information documented in participant files, including documented or self-reported admissions (if admitted to another institution, this information may be unknown). Additionally, the study was conducted at a district-level hospital, which means that a sicker participant population is possible. The data were captured during the coronavirus disease 2019 (COVID-19) pandemic, which may have posed an independent risk to morbidity, compliance and follow-up. Because of the novelty of DTG in SA, the duration of participant exposure to EFV-based regimens in our study sample was significantly longer than that of DTG, with an average exposure to EFV of 69 months and to DTG of 24 months. Additionally, we acknowledge that CrCl may overestimate renal function, particularly in HIV populations with low muscle mass. This consideration should be taken into account when evaluating the study’s findings.

The design was well suited for identifying patterns or associations between ART regimens and metabolic outcomes. The study setting of KZN, the global highest HIV prevalence, provides a rich context for examining the effects of ART across different demographic groups, offering insights into population-specific responses.

### Recommendations

Despite the relatively short duration of DTG use in the population being studied, there is a notably higher incidence of newly diagnosed chronic conditions emerging among participants in the Switch group who previously had TEE exposure. These resulted in a statistically significant increase in hospitalisations related to metabolic disease and associated complications thereof. The study also emphasises the need to Switch from the existing EFV-based regimens, which showed a significant increase in HIV-associated complications and hospitalisations, highlighting the effect of DTG in immune reconstitution. Considering this need to Switch from EFV, DTG appears to be the best option. This study, however, sheds light on the need to find ways to control and try to limit the metabolic side effects of DTG in the population subset with pre-existing comorbid disease, especially among populations with underlying associated risk factors. Further studies assessing the impact of lifestyle modification and anti-hypertensive and lipid-lowering therapy on these participants will be of benefit in evaluating this. This will play an integral role in participant care by recommending additional measures that can be added to participant care and counselling with the aim of decreasing adverse effects associated with ART.

Human immunodeficiency virus and NCDs pose significant burdens in SA. Cardiovascular disease is one of the leading non-acquired immunodeficiency syndrome (AIDS)-defining causes of death among PLHIV.^2^ A highly effective drug in suppressing the HIV VL that is cheap and being rolled out en masse may significantly benefit PLHIV. Still, it may have long-term adverse cardiovascular and metabolic outcomes, and the increasing burden of NCDs may offset the gains made by managing HIV.

## Conclusion

This study highlights key clinical implications of using DTG in SA’s HIV programme, especially as DTG is adopted in various ART regimens. While DTG regimens show immunological benefits, such as CD4 count increases in TLD and Switch groups, concerns arise about metabolic and renal outcomes, mainly among patients switching from efavirenz-based regimens. The Switch group experienced more new and worsening comorbidities, with renal function deterioration crossing into pathological ranges and persisting at 12 months. The TLD group showed metabolic stability, including weight neutrality and better glycaemic and hypertensive control, indicating a safer profile when initiated in ART-naïve individuals. Despite higher HIV-related hospital admissions in the TEE group, the Switch group had more comorbidity-related admissions. These findings emphasise the need for stratified monitoring and proactive NCD management, especially for those transitioning from legacy regimens. As the PLHIV population ages, integrating NCD care into ART programmes is vital to improve long-term health outcomes.

## References

[1] Republic of South Africa, National Department of Health, Republic of South Africa. ART clinical guidelines for the management of HIV in adults, pregnancy, adolescents, children, infants and neonates. Pretoria: National Department of Health, Republic of South Africa; 2023.

[2] Sebilo M, Ledibane NRT, Takuva S. Incidence of cardiometabolic diseases in a Lesotho HIV cohort: Evidence for policy decision-making. South Afr J HIV Med. 2021;22(1): 1246. 10.4102/sajhivmed.v22i1.1246 34230861 PMC8252144

[3] Hailu W, Tesfaye T, Tadesse A. Hyperglycemia after dolutegravir-based antiretroviral therapy. Int Med Case Rep J. 2021;14:503–507. 10.2147/IMCRJ.S323233 34349567 PMC8326784

[4] Quercia R, Roberts J, Martin-Carpenter L, Zala C. Comparative changes of lipid levels in treatment-naive, HIV-1-infected adults treated with dolutegravir vs. efavirenz, raltegravir, and ritonavir-boosted darunavir-based regimens over 48 weeks. Clin Drug Investig. 2015;35(3):211–219. 10.1007/s40261-014-0266-2 PMC4335094 25637061

[5] Sinxadi PZ, McIlleron HM, Dave JA, et al. Plasma efavirenz concentrations are associated with lipid and glucose concentrations. Medicine (Baltimore). 2016;95(2):e2385. 10.1097/MD.0000000000002385 26765416 PMC4718242

[6] Eyawo O, Brockman G, Goldsmith CH, et al. Risk of myocardial infarction among people living with HIV: An updated systematic review and meta-analysis. BMJ Open. 2019;9(9):e025874. 10.1136/bmjopen-2018-025874 PMC6773316 31551371

[7] Hill AM, Mitchell N, Hughes S, Pozniak AL. Risks of cardiovascular or central nervous system adverse events and immune reconstitution inflammatory syndrome, for dolutegravir versus other antiretrovirals: Meta-analysis of randomized trials. Curr Opin HIV AIDS. 2018;13(2):102–111. 10.1097/COH.0000000000000445 29278532

[8] Rebeiro PF, Emond B, Rossi C, et al. Incidence of cardiometabolic outcomes among people living with HIV-1 initiated on integrase strand transfer inhibitor versus non-integrase strand transfer inhibitor antiretroviral therapies: A retrospective analysis of insurance claims in the United States. J Int AIDS Soc. 2023;26(6):e26123. 10.1002/jia2.26123 37306118 PMC10258864

[9] Norwood J, Turner M, Bofill C, et al. Brief report: Weight gain in persons with HIV switched from efavirenz-based to integrase strand transfer inhibitor-based regimens. J Acquir Immune Defic Syndr. 2017;76(5):527–531. 10.1097/QAI.0000000000001525 28825943 PMC5680113

[10] Griesel R, Maartens G, Chirehwa M, et al. CYP2B6 genotype and weight gain differences between dolutegravir and efavirenz. Clin Infect Dis. 2021;73(11):e3902–e3909. 10.1093/cid/ciab804 32960272 PMC8653639

[11] Koteff J, Borland J, Chen S, et al. A phase 1 study to evaluate the effect of dolutegravir on renal function via measurement of iohexol and para-aminohippurate clearance in healthy subjects. Br J Clin Pharmacol. 2013;75(4):990–996. 10.1111/j.1365-2125.2012.04440.x 22905856 PMC3612717

[12] Weller S, Borland J, Chen S, et al. Pharmacokinetics of dolutegravir in HIV-seronegative subjects with severe renal impairment. Eur J Clin Pharmacol. 2014;70(1):29–35. 10.1007/s00228-013-1590-9 24096683 PMC3889630

[13] Lu L, Li X, Liu X, et al. Comparison of renal function biomarkers of serum creatinine and cystatin C in HIV-infected people on dolutegravir-containing therapy. Infect Drug Resist. 2022;15:1695–1706. 10.2147/IDR.S347054 35422637 PMC9005235

[14] Barata R, Marques da Costa B, Navarro D, et al. Acute interstitial nephritis due to dolutegravir: The first case reported. Nefrologia (Engl Ed). 2023;43(3):370–373. 10.1016/j.nefroe.2022.11.012 36437200

[15] Martínez-Serra A, De Lazzari E, Berrocal L, et al. Clinical use and effectiveness of dolutegravir and lamivudine: A long-term, real-world, retrospective study. J Antimicrob Chemother. 2023;78(8):1955–1962. 10.1093/jac/dkad189 37311224

[16] Gebremedhin T, Aynalem M, Adem M, Geremew D, Aleka Y, Kiflie A. Dolutegravir based therapy showed CD4+T cell count recovery and viral load suppression among ART naïve people living with HIV AIDS: A pilot evaluation. Sci Rep. 2024;14(1): 3297. 10.1038/s41598-024-53282-y 38331983 PMC10853173

[17] Zhong M, Li M, Qi M, et al. A retrospective clinical study of dolutegravir- versus efavirenz-based regimen in treatment-naïve patients with advanced HIV infection in Nanjing, China. Front Immunol. 2023;13:1033098. 10.3389/fimmu.2022.1033098 36700216 PMC9868135

[18] National Department of Health, Republic of South Africa. Standard Treatment Guidelines and Essential Medicines List for South Africa: Primary Healthcare Level, 2023 Edition. Pretoria: National Department of Health; 2023.

[19] National Department of Health, Republic of South Africa. Standard Treatment Guidelines and Essential Medicines List for South Africa: Primary Healthcare Level. 7th ed. Pretoria: National Department of Health; 2020.

[20] Romo ML, Patel RC, Edwards JK, et al. Disparities in dolutegravir uptake affecting females of reproductive age with HIV in low- and middle-income countries after initial concerns about teratogenicity: An observational study. Ann Intern Med. 2022;175(1):84–94. 10.7326/M21-3037 34843382 PMC8808594

[21] Mbabazi L, Nabaggala MS, Kiwanuka S, et al. Factors associated with uptake of contraceptives among HIV positive women on dolutegravir based anti-retroviral treatment-a cross sectional survey in urban Uganda. BMC Womens Health. 2022;22(1):262. 10.1186/s12905-022-01842-7 35761248 PMC9238171

[22] Summers NA, Lahiri CD, Angert CD, et al. Metabolic changes associated with the use of integrase strand transfer inhibitors among virally controlled women. J Acquir Immune Defic Syndr. 2020;85(3):355–362. https://doi.org10.1097/QAI.0000000000002447 33060420 10.1097/QAI.0000000000002447 PMC7577246

